# Mid-lumbar (L3) epidural stimulation effects on bladder and external urethral sphincter in non-injured and chronically transected urethane-anesthetized rats

**DOI:** 10.1038/s41598-023-39388-9

**Published:** 2023-07-28

**Authors:** Daniel Medina-Aguiñaga, Robert F. Hoey, Natasha L. Wilkins, Beatrice Ugiliweneza, Jason Fell, Susan J. Harkema, Charles H. Hubscher

**Affiliations:** 1 Department of Anatomical Sciences and Neurobiology, University of Louisville School of Medicine, 511 S. Floyd St., MDR, Room 111, Louisville, KY 40202 USA; 2 Department of Neurological Surgery, University of Louisville School of Medicine, Louisville, KY USA; 3 Kentucky Spinal Cord Injury Research Center, University of Louisville, Louisville, KY USA; 4 Department of Health Management and Systems Science, School of Public Health and Information Science, University of Louisville, Louisville, KY USA; 5 Physical Medicine and Rehabilitation Department, MetroHealth Rehabilitation Institute of Ohio, Cleveland, OH USA; 6 Physical Medicine and Rehabilitiation Department, Case Western Reserve University School of Medicine, Cleveland, OH USA

**Keywords:** Spinal cord injury, Bladder

## Abstract

Recent pre-clinical and clinical spinal cord epidural stimulation (scES) experiments specifically targeting the thoracolumbar and lumbosacral circuitries mediating lower urinary tract (LUT) function have shown improvements in storage, detrusor pressure, and emptying. With the existence of a lumbar spinal coordinating center in rats that is involved with external urethral sphincter (EUS) functionality during micturition, the mid-lumbar spinal cord (specifically L3) was targeted in the current study with scES to determine if the EUS and thus the void pattern could be modulated, using both intact and chronic complete spinal cord injured female rats under urethane anesthesia. L3 scES at select frequencies and intensities of stimulation produced a reduction in void volumes and EUS burst duration in intact rats. After chronic transection, three different subgroups of LUT dysfunction were identified and the response to L3 scES promoted different cystometry outcomes, including changes in EUS bursting. The current findings suggest that scES at the L3 level can generate functional neuromodulation of both the urinary bladder and the EUS in intact and SCI rats to enhance voiding in a variety of clinical scenarios.

## Introduction

Lower urinary tract (LUT) dysfunction is one of the primary concerns of people living with spinal cord injury^1–3^ (SCI), as it is one of the principal causes of re-hospitalization after SCI and a major source of morbidity and mortality^4^. After SCI, neurogenic bladder develops, characterized by storage and voiding dysfunction with detrusor sphincter dyssynergia (DSD) as the most significant source of LUT complications. DSD is when bladder contractions are coincident with urethral sphincter contractions, causing the pressure inside the LUT to rise to unsafe levels. Chronic elevation of vesical pressure can lead to LUT and kidney damage.

Daily management for LUT dysfunction includes a combination of catheterization (either self-intermittent or suprapubic) and pharmacological approaches, but does not restore lost functionality, including voluntary control of emptying. Pharmacological approaches primarily target hyper-reflexive bladder activity that occurs after injury, which leads to incontinence, small storage volumes, and increased vesicle pressure. The current management strategy is not sufficient as frequent bladder catheterization leads to a higher risk of bladder infection, which then drives the high rate of rehospitalization in SCI individuals^5^.

Therapies targeting LUT function with neuromodulation that utilize electrical stimulation of peripheral nerves (tibial, saphenous, pudendal, and the dorsal genital nerve), sacral nerves, and sacral anterior roots combined with posterior root rhizotomy have all shown positive effects on bladder storage and emptying after SCI^6–11^. However, each technique has limitations, which impacts their utility for chronic treatment and the willingness of people living with SCI to adopt these LUT treatments^5,12–15^. A more recent approach, spinal cord epidural stimulation (scES; use of an electrode array implanted on the surface of the dura mater to provide electrical stimulation), that has been examined in multiple individuals living with SCI for stepping, voluntary movement, and cardiovascular control, has shown off-target improvements in LUT function^9,10,13,14^.

More recent pre-clinical and clinical scES experiments directly targeting the thoracolumbar and lumbosacral circuitries mediating LUT function have shown improvements in storage, detrusor pressure, and emptying^9,10,15,16^. However, although targeting L6/S1 (parasympathetic level) and T13/L1 (sympathetic level) via scES in the urethane-anesthetized SCI rodent model during cystometry can lead to changes in inter-contractile interval (ICI; time between two voids) and detrusor activation, it does not alter the incidence of DSD. The lack of coordination between the external urethral sphincter (EUS) and detrusor muscle, resulting in low-volume voids, high bladder pressure, and an increased risk of autonomic dysreflexia^17–19^, may need to be ameliorated by targeting the bladder and EUS circuitries with scES simultaneously. Experimental data supports the existence of a lumbar spinal coordinating center (LSCC) at L3 in rats that modulates EUS activation during micturition. The LSCC has projections to both L6/S1 and T13/L1 to coordinate activation of the EUS and detrusor^20,21^. In the current pre-clinical study, the L3 was targeted with scES to examine whether the EUS could be modulated under intact and chronic complete SCI conditions.

## Methods

### Animal groupings

All animal procedures conformed to NIH guidelines and were reviewed and approved by the Institutional Animal Use and Care Committee at the University of Louisville, School of Medicine. All experiments were performed in accordance with relevant guidelines and regulations and follow recommendations in the ARRIVE guidelines. A total of 18 female Wistar rats were used for this study. Half the animals (n = 9) received a complete spinal cord transection at the T9 spinal level, whereas a second group (n = 9 surgical shams) had a laminectomy but no SCI.

### SCI’s

Under ketamine/xylazine anesthesia (80/10 mg/kg, intraperitoneally) and aseptic conditions, animals underwent a dorsal incision and laminectomy at T8 , as previously described^15,16^. T9-level transections were performed with micro-scissors. Gel foam was inserted into the lesion site prior to closure of the dorsal muscle with 4–0 suture (Ethicon) and skin with wound clips (Mikrotek, 9 mm autoclip). After surgery, the rats receive daily manual bladder emptying 3/day until voiding reflexively. Pain medication (Meloxicam,1/day for 3 days); and antibiotics (Gentafuse, gentamiacin, SC, 1/day for 3 days; Penject, penicilin G, SC, 1/day for 3 days) were provided per our laboratories standard protocol^15,16^.

### Terminal mapping study preparation

Animals were initially anesthetized with Isoflurane (5% induction, and 2% maintenance) and placed in a supine position on a water-heated pad (Gaymar) to maintain body temperature. A sagittal skin incision (~ 1 cm) was made lateral to the trachea and the tissues were blunt dissected for access to the jugular vein. A PE-60 (Intramedic, Clay Adams) jugular catheter was implanted by isolating the vessel from the connective tissue, tying of the rostral end with 4–0 silk suture, making a small incision in the vein with micro-scissors, inserting the polyethylene tubing and securing the caudal end of the vessel around the catheter with silk suture. Both, the rostral and caudal ligatures were tied around the catheter to ensure orientation and structural integrity. Anesthesia was switched from isoflurane to IV urethane (1.2 g/kg) gradually by reducing the isoflurane percentage and slowly infusing the urethane over a 10–15 min period and maintaining continuous surgical depth of anesthesia (measured by corneal response and respiration rate). The IV route of urethane administration was specifically selected for this study as intraperitoneal administration can have a suppressive effect on bladder function. IV administration also avoids variability in anesthetic depth typically seen with subcutaneous administration. Supplemental anesthesia was given to animals (IV, 0.05 ml increments) as needed. Once the full dose was provided, the trachea was dissected and partially opened between the 6th and 7th tracheal rings. A polyethylene tubing was placed into the trachea to assure airway flow. Lastly, the skin was closed with 4–0 silk suture.

A bladder catheter, EUS and external anal sphincter (EAS) electrodes were implanted following previously published protocols^15,16^ . Briefly, the catheter and wiring were implanted such that they were externalized via a scapular incision through a subcutaneous tunnel from the abdomen. The abdominopelvic cavity was opened, and the bladder exposed. The bladder dome was punctured with an 18-gauge needle and the catheter (PE-60 tubing with a heat-flared end) inserted into the lumen and secured with a collar of silk suture (4–0). The EUS muscle, located below the bladder neck and rostral to the pelvic symphysis and inguinal ligaments, was then exposed and implanted with two thin wires (A-M Systems, 0.002″ diameter, stainless steel) with the tip cut on a severe angle and a loop formed 2 cm before the distal end of the wire. The loops were sutured (6–0, Ethicon) to the inguinal ligaments for stabilization of the electrode implant and the stripped tip of the electrodes inserted into the muscle bilaterally. A third electrode was inserted into the fascia of the abdominal muscles as a reference. The catheter and electrodes were tunneled subcutaneously to the back of the neck. For the EAS, the electrode tips were stripped, a small hook was made and the tip pierced directly into the muscle (wires remained externalized).

A multi-electrode epidural stimulation array (Specify 5–6–5, Medtronic, Minneapolis, MN) modified for use of one electrode row (5 contacts, each electrode contact is 2 mm W × 4 mm L with 4 mm between contacts) was used to perform the stimulation during terminal testing procedures as previously described^15,16^. After a quadruple laminectomy, two electrodes were placed rostro-caudal on the epidural surface, one over L3 cord as cathode (stimulation) with the other above L2 as anode (ground). Each electrode covered the mediolateral spinal cord up to the dorsal root entry zone on both sides. The array was then secured mechanically by gently suturing the muscle layer together over the implant.

### Mapping procedure

After instrumentation was complete (described above), the bladder catheter was connected to a pump for saline infusion via a three-way connector (Braintree Scientific, Braintree, MA, USA) and a pressure transducer (World Precision Instruments [WPI, LLC]; Sarasota, FL, USA) for bladder pressure recording. The three EUS-EMG electrodes were connected to a differential amplifier (A-M Systems, Sequim, WA, USA).

The animal was placed in a prone position on a surgical table and the urine collected and measured using two different methodologies. In the early phase of the study, the voided volume of fluid excreted during each voiding cycle was measured using a balance (Ohaus, Scout) with a Petri dish to collect the fluid. Balance values were transmitted to the computer via a USB device interface cable (RS232) and software (Serial Port Data Collection, SPDC, Ohaus, V2.01; https://us.ohaus.com/en-us/support/software-and-drivers) to Excel where formulas were used to calculate the volume per micturition. Later in the study, a second more user friendly method was adopted, which consisted of a container coupled to a weight transducer (WPI, LLC) placed under the rat for urine collection and measurement. The signal was amplified (WPI Transbridge 4 M amplifier) and then exported directly into the data file (Spike 2, V8.21) via a CED Mircro3 1401 unit. The updated second setup allows for direct measurement of the expelled fluid in the same file, at the same time as all the other data traces.

All instrumentation traces were acquired using Cambridge Electronic Device (CED Micro3 1410) and Spike 2 (V8.21) software. Bladder pressure (CMG) and electromyographic data (EUS and EAS EMG) signals were amplified with a 4-channel pressure amplifier (WPI Transbridge 4 M amplifier) and a 4-channel differential AC amplifier (AM-Systems, model 1700) respectively. Pressure probes for anorectal manometry (ARM; inserted to 2 and 10 cm positions from anal verge) (Millar SPR-524) were detected by a Millar unit and recorded^22^.

The infusion pump for filling cystometry (CMG) was then started at a constant fill rate of 0.25 ml/min^15,16^. Per published protocols, initial bladder pressure responses to the physiological saline infusion were observed during an acclimation period until the fill-void cycle had consistent performance and 5 cycles were recorded as baseline. After the baseline parameters were collected, scES was applied (ON) followed by a non-stimulation period (OFF) at each parameter combination. The combinations included five different frequencies (5, 10, 30, 45, and 60 Hz) and up to five intensities (50, 75, 100, 150, 300 µA). Note that once a visualized hindlimb movement occurred, no further increase in intensity was tested as the goal is to map for sub-motor threshold scES parameters for LUT-related functions. All combinations were tested using the following parameters per previous studies^15,16^: 1 ms pulse duration, no delay, 1 train per second, and a 500 ms train duration. Either of two different parameter sequences were applied (randomly selected), both starting with 5 Hz 50 µA. For one sequence, the frequency was modified first at each fixed intensity, whereas for the other, intensity was varied first at each fixed frequency, until all combinations were applied. Void to void stimulation was used throughout the mapping procedure- one micturition cycle would have epidural stimulation on while the next would have no stimulation. If the stimulation resulted in the prevention of a void despite a full bladder, the stimulation would remain ON until the animal entered a state of overflow incontinence (leak of multiple drops without detrusor contraction). After overflow incontinence was reached, the stimulus would be turned off until micturition void cycles returned to baseline inter-contractile interval (ICI). EAS EMG activity and peristaltic activity in the rectum (2 cm ARM probe) and distal colon (10 cm ARM probe) including any scES-induced effects (activation or suppression) were also recorded throughout the study simultaneously (these data will be published elsewhere).

### Statistical analysis

The CMG parameters evaluated included ICI, voided volume, burst duration (duration of the phasic electrical activity of the EUS during a single void) and calculated in–out ratio r = ($\frac{ml\, saline\, voided}{ml\, saline\, infused}$). Data was analyzed using a one-way repeated measures ANOVA or a mixed effects analysis. Because of different sensitivities to scES, some data sets did not have the higher amplitude stimulation combinations. Therefore, a mixed model was used, followed by Šídák's multiple comparisons test to evaluate significance between baseline and scES combinations. The significance level was set at *p* ≤ 0.05 (Prism 7, GraphPad, San Diego, CA). Data are presented as mean ± SEM. Any non-parametric data was analyzed with a Friedman’s test followed by Dunn’s multiple comparisons test.

## Results

The current data identifies the LUT responses to scES at L3 using the modified electrode array in 15 of 18 sham and transected female rats 6 weeks post-injury. Of the three rats that had no data collected, one intact and one SCI rat overdosed from anesthesia prior to the start of the terminal study, and one had a bladder catheter issue. For the 15 rats that completed the study, electrode location was verified with markings and post-perfusion dissection and identification of the dorsal roots. Individual data for 8 intact and 7 transected female rats were averaged together for the baseline parameters, and by group for each of the parameter combinations tested. Note that not all injured animals received the highest intensity of stimulation (300 µA) due to hindlimb movements.

### Baseline CMG

**Figure 1:**
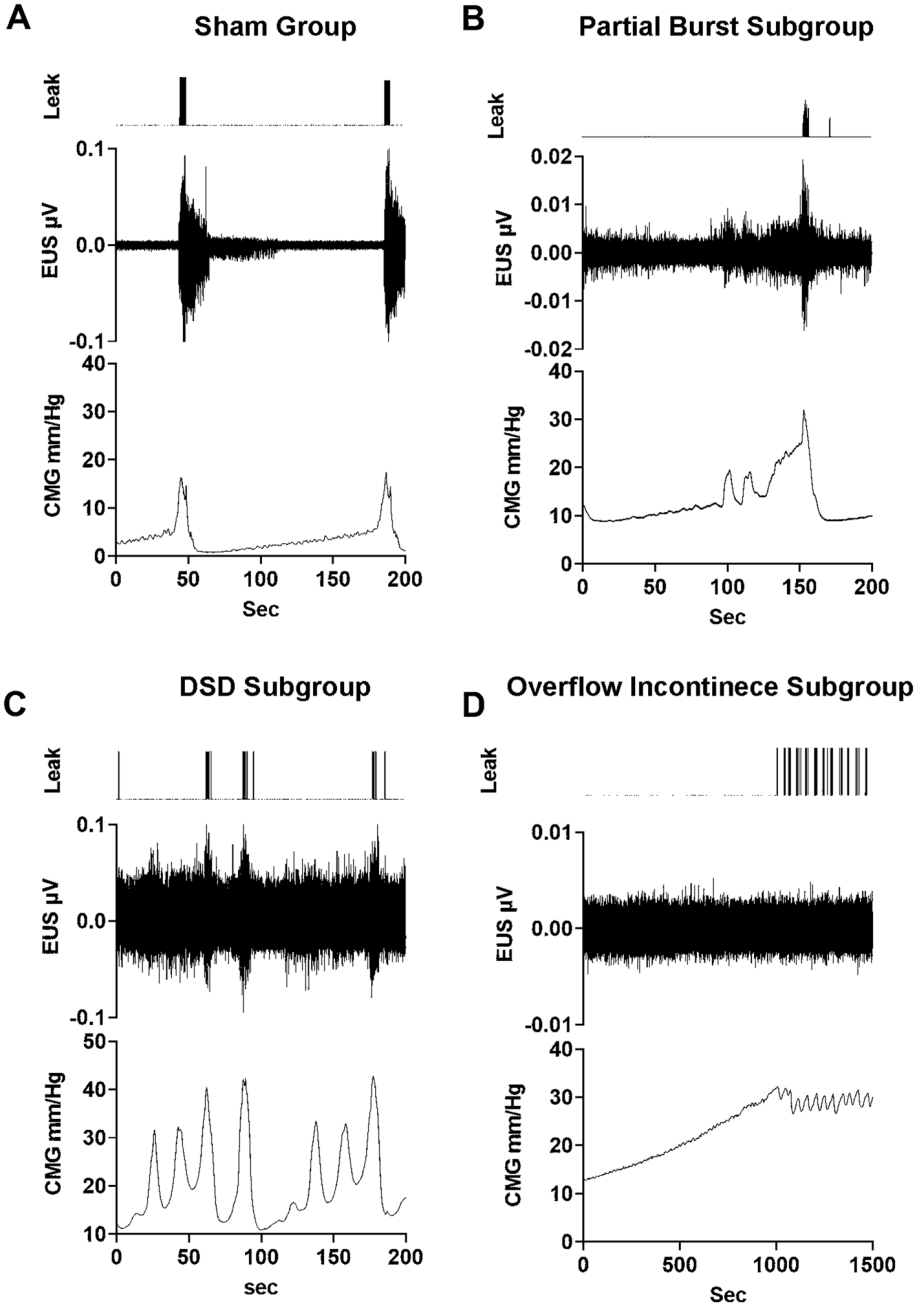
*Examples of cystometric and EUS electromyographic (CMG-EMG) activity during fill-void cycle(s) in SHAM and SCI female rats*. The CMG-EMG in sham animals (**A**) shows a typical fill-void cycle with low amplitude intra vesical pressure (IVP) and low tonic activity of the EUS during the filling phase, and the presence of high frequency oscillations (HFOs), phasic activity of the EUS and multiple drops during the expulsion phase (leak markings from drop collections shown at the top of each recording). After 6 weeks of injury, three different fill-void cycle patterns were found (**B**–**D**). Patterns included partial bursting (**B**), detrusor sphincter dyssynergia (DSD, **C**) and overflow incontinence (**D**). The partial burst pattern (n = 1 of 7 transected rats) shows increased baseline pressure, some low amplitude non-voiding contractions (NVC) and increased tonic EUS EMG activity during the filling phase. During the void, the animal preserved some phasic activation of the EUS, HFO and adequate void volume. The animals in the DSD group (n = 4 of 7) show increased baseline pressure, many high amplitude NVC’s and increased tonic EUS activity during the filling phase. During the void phase, the bladder pressure is high prior to expulsion of urine, tonic activity of EUS is present during expulsion, and there is a reduction in void volume. The overflow incontinence group (n = 2 of 7) presented with a constant EUS activation and increasing bladder pressure during filling until overflow incontinence at capacity. Note that expelled drops were synchronized with small contractile activity of the detrusor.

**Figure 2:**
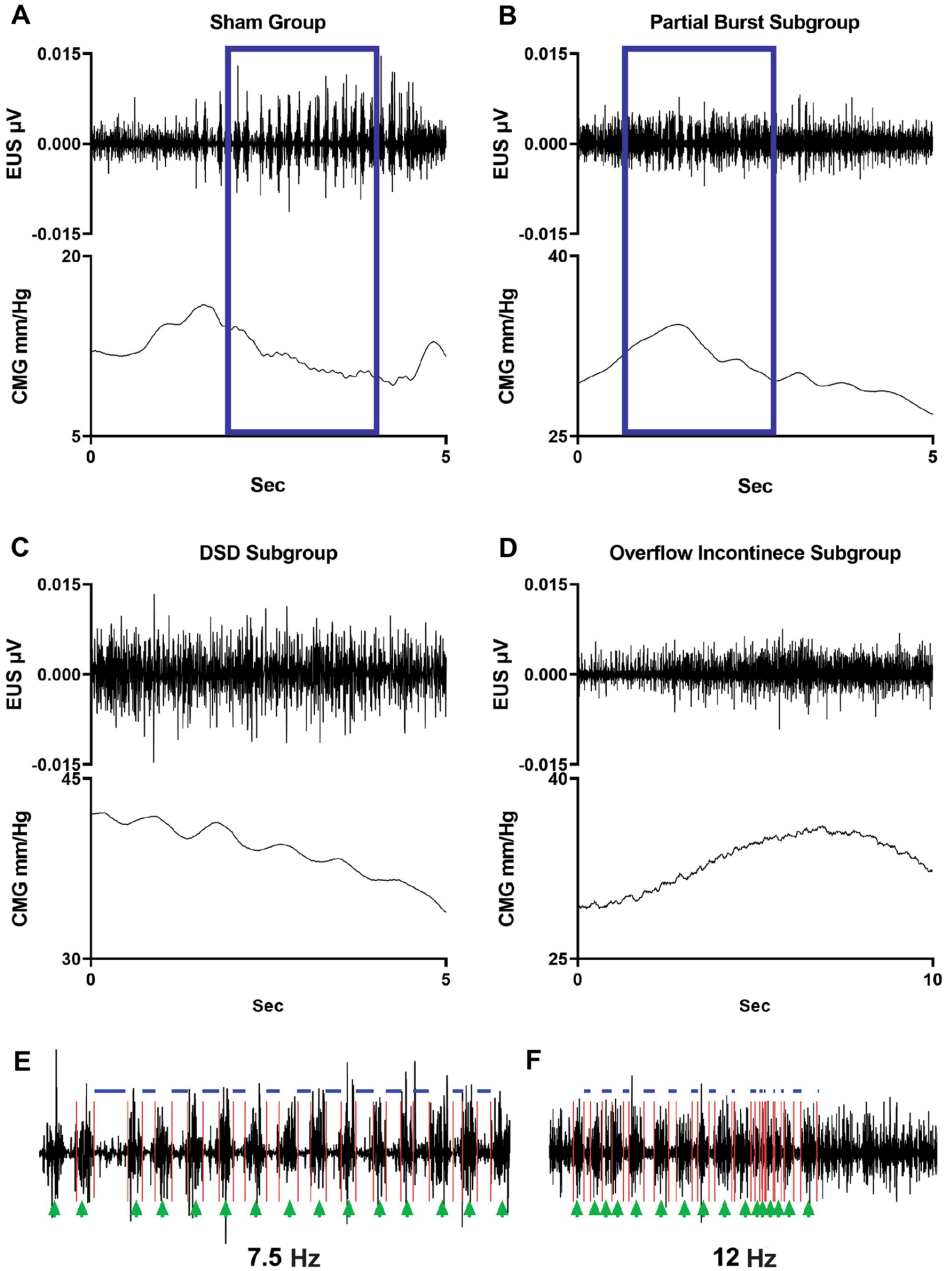
*External urethral sphincter (EUS) electrical activity during void in non-injured and chronically transected rats.* In Sham animals (**A**), during the isovolumetric contraction of the bladder, the EUS displayed tonic electrical activity followed by phasic activation causing the opening and closure of the urethra, a drop in urinary bladder pressure and the flow of urine. Twelve weeks post injury, the animals show different patterns of urinary dysfunction. In (**B**), the partial phasic activity of the EUS during the micturition is shown. The DSD (**C**, n = 4) pattern included those animals who exhibited tonic activity of the EUS during the void. The last group (n = 2) was characterized by a mixed dysfunction of the urinary bladder and the EUS, which includes a hypo-contractile urinary bladder, tonic activation of the EUS, and overflow incontinence. The blue box in (**A** and **B**) are magnified in (**E** and **F**). In shams, the phasic activity during micturition is characterized by a regular (7.5 Hz) burst pattern and consistent active (green arrows) and inactive (blue lines) periods. After SCI, an increment in the burst pattern (12 Hz) as well as a decrement in the durations of the periods of inactivity of the EUS during bursting is evident.

Sham animals had consistent fill-void cycles that showed typical CMG pressure curves and EUS EMG bursting during pre-mapping baseline micturition cycles (Figs. 1A and 2A, respectively). The storage fill phase was characterized by low bladder pressure and reduced tonic activity of the EUS (Fig. 1A). During the emptying phase, CMG recordings from intact animals showed low urethral opening thresholds, the presence of high frequency oscillations (HFO’s) and EUS phasic bursting activity (Fig. 2A, E), and high void volumes. The EUS phasic activity is characterized by repeated bouts of high frequency activity followed by a short period of electric inactivity between bouts (Fig. 2E). This bursting pattern of EUS activity found in sham animals is characteristic of that found in spinally intact animals in the literature, including our previously published reports^23,24^. For shams, the mean voided volume was 0.53 ± 0.06 ml, ICI duration 109.1 ± 40.9 s, in–out ratio 0.98 ± 0.22 and, 3.2 ± 0.33 s burst duration (Supplemental Table 1).

At six weeks post-SCI during baseline CMG recordings, all rats had neurogenic urinary dysfunction, which affected both bladder contractility (Fig. 1B–D) and EUS performance (Fig. 2B–D). Although all animals received a complete transection, it was possible to distinguish three different patterns of urinary dysfunction. The first subgroup, labeled as partial burst (n = 1 of 7), was characterized by regular fill-void cycles (ICI = 159.38 ± 14.2 s), the presence of non-voiding contractions (NVCs, 3 ± 1.09 NVC/cycle), and increased tonic activity during the filling phase (Fig. 1B). The voiding phase was characterized by low amplitude urethral opening pressure (31.9 ± 0.93 mmHg) (Fig. 1B), adequate voided volume (0.66 ± 0.04 ml), and an in–out ratio of 1.01 ± 0.12. This animal presented with impaired EUS bursting activity with a low number of irregular bursts (7.5 ± 0.86 burst/void), and reduced duration of inactivity between each burst (2.47 ± 0.86, Fig. 2B and F, Supplemental Table 1). The second group, which presented as a DSD pattern of void activity (n = 4 of 7), was characterized by regular fill-void cycles (ICI = 99.5 ± 28.83 s), the presence of high amplitude non-voiding contractions (1.4 ± 1.2 NVC/micturition cycle) and increased tonic EUS electrical activity during the filling phase (Fig. 1C). During voiding, these animals had high urethral opening pressures (37.6 ± 4.34 mmHg), similar voided volumes (0.53 ± 0.06 ml), and the presence of tonic EUS activity (lack of bursting activity) during micturition (Fig. 2C, Supplemental Table 1). The last group of animals displayed overflow urinary incontinence (n = 2) characterized by constant dripping upon reaching capacity during the first fill cycle (drops with every small contraction of the detrusor). These animals showed continuous fluid expulsion at an elevated (compared with SHAM) urethral opening pressure (34.34 ± 2.94 mmHg), small bladder contraction amplitude (6.2 ± 2.62 mmHg), short ICI (29.9 ± 18.72 s), less than 4 drops per void, and minimal changes in the amplitude of EUS activation (Figs. 1D and 2D, Supplemental Table 1).

### CMG with scES

**Figure 3:**
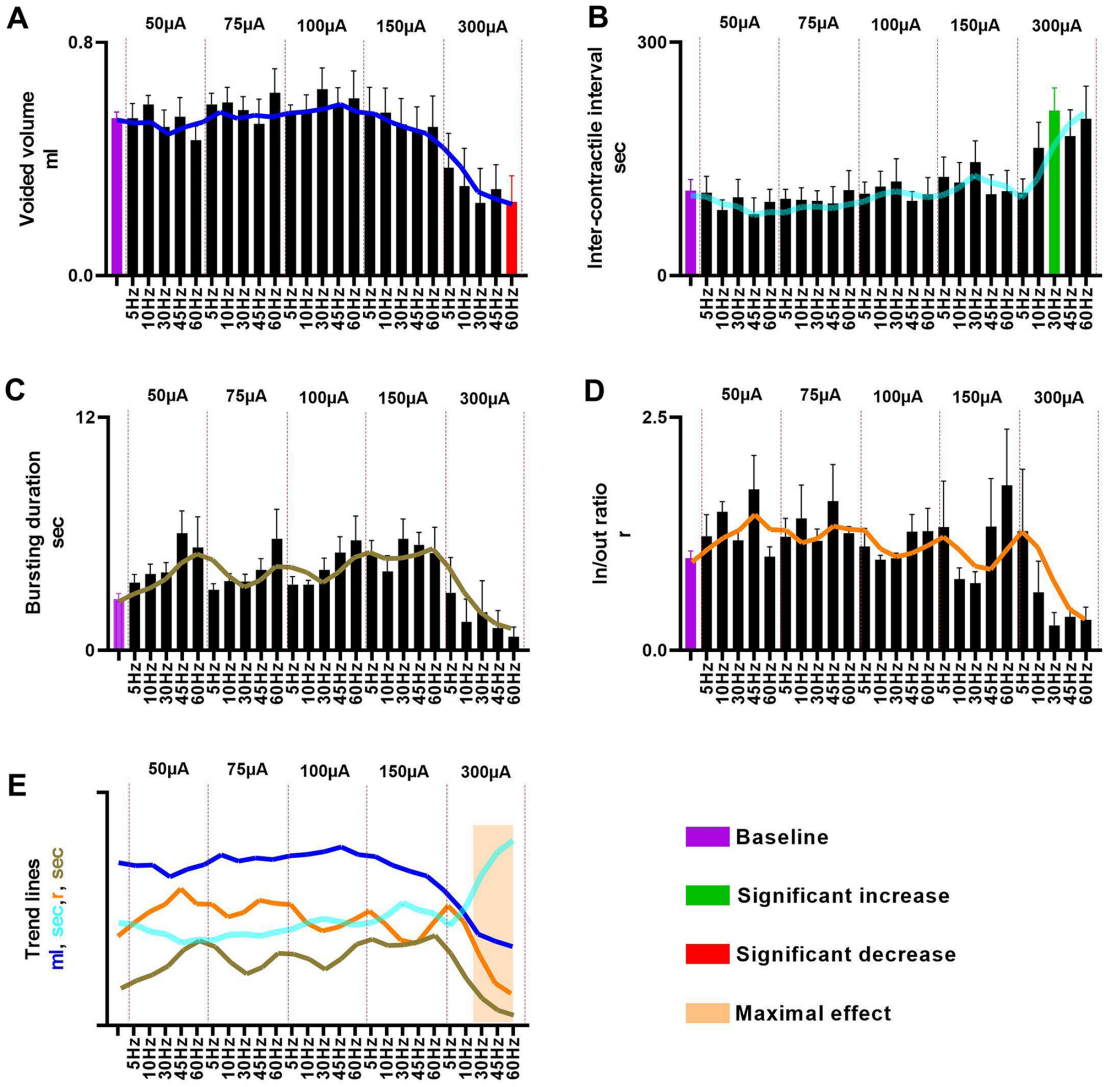
*Parameter Summary.* Overview of the combination of all frequencies and intensities of scES at L3 on the cystometric parameters averaged from eight sham female rats: green and red bars represent significant changes relative to baseline (purple; no scES). The colored lines over the bars (graphed together in **E**) represents the trend for the four parameters graphed in (**A**–**D**). High frequencies and intensities significantly reduced the voided volume (*p* = 0.0283; **A**), significantly increased the ICI (*p* = 0.0488; **B**), reduced the bursting duration (**C**), as well as the void efficiency (**D**). The combined trend line plot in E indicates the optimal sets of scES parameters (tan shadow: highest ICI re storage; disruption of EUS bursting re shorter burst duration translating to reduction in void volume and thus in/out ratio).

**Figure 4:**
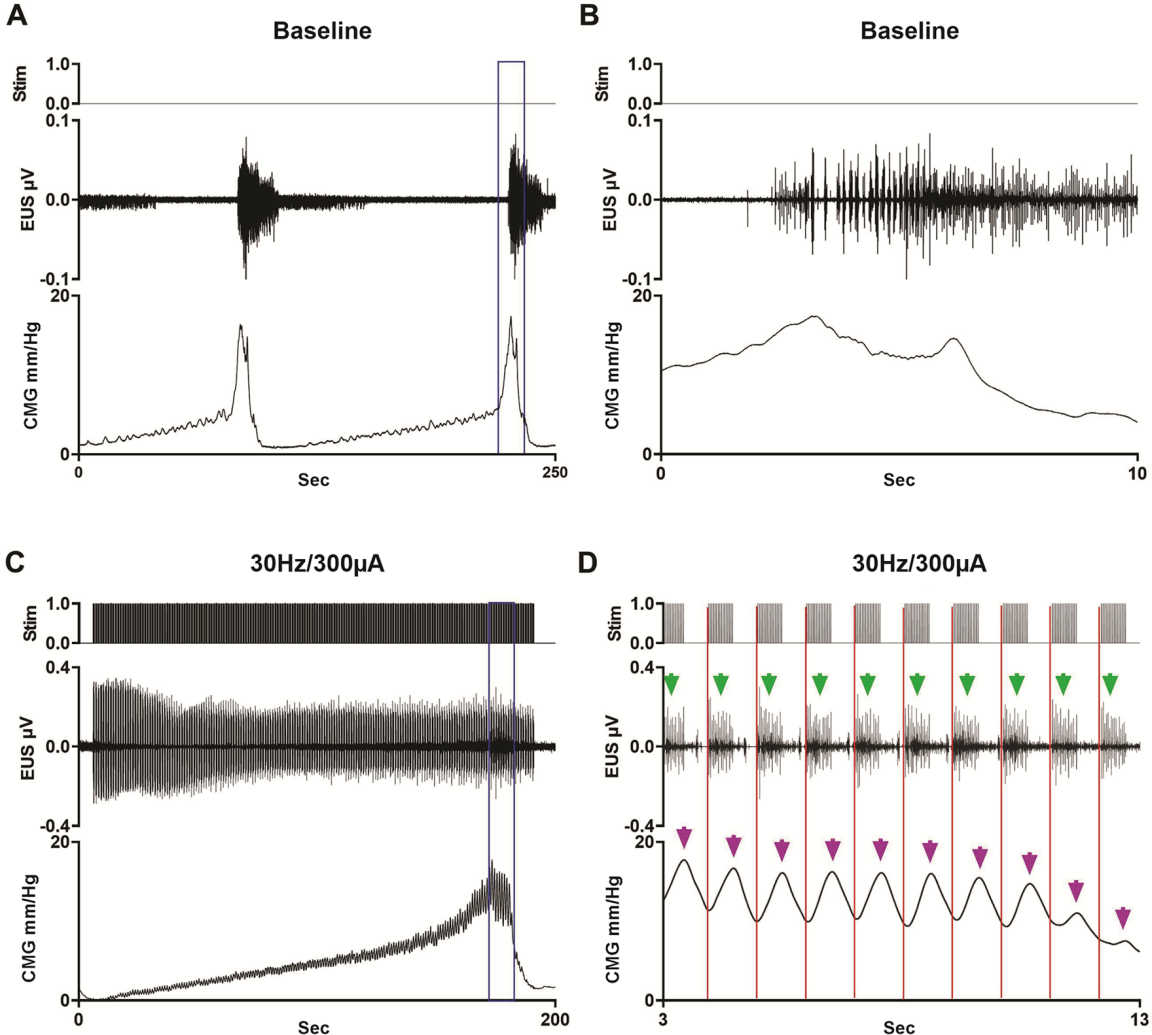
*L3 scES Typical Example in Shams.* The application of scES at 30 Hz/300 µA on L3 in a non-injured female rat increased the inter-contractile interval and disrupted the phasic EUS activity (**A** baseline versus **C** with scES; magnifications of boxed region in **B** and **D**, respectively). Note the temporal correlation between the stimuli artifacts (red lines; artifact within EUS EMG recording), the EUS activity (green arrows) and rise in bladder pressure (purple arrows).

The combined CMG/EUS EMG data from sham rats indicate that 300μA scES at spinal level L3 significantly reduced void volume at 60 Hz (Fig. 3A) and significantly increased ICI at 30 Hz (Fig. 3B). Additionally, frequency dependent fluctuations in EUS burst duration and in/out ratio was found (Figs. 3C, D), but not to the level of significance relative to baseline. A summary of the data (Fig. 3E) indicates several trends. The EUS bursting activity was particularly affected by scES at the L3 level at 300μA (typical example in Fig. 4). At that high intensity there was either a reduction in the duration of EUS activity or a complete disruption in the endogenous bursting pattern wherein the EUS showed a massive rhythmic activation mimicking the trains of stimulation (Figs. 3C and 4D). The scES-induced EUS contractions were reflected in the urinary bladder by rapid, rhythmic, and high amplitude increases in pressure, which is usually seen during the voiding phase of micturition (HFOs; Fig. 4B and D).

**Figure 5:**
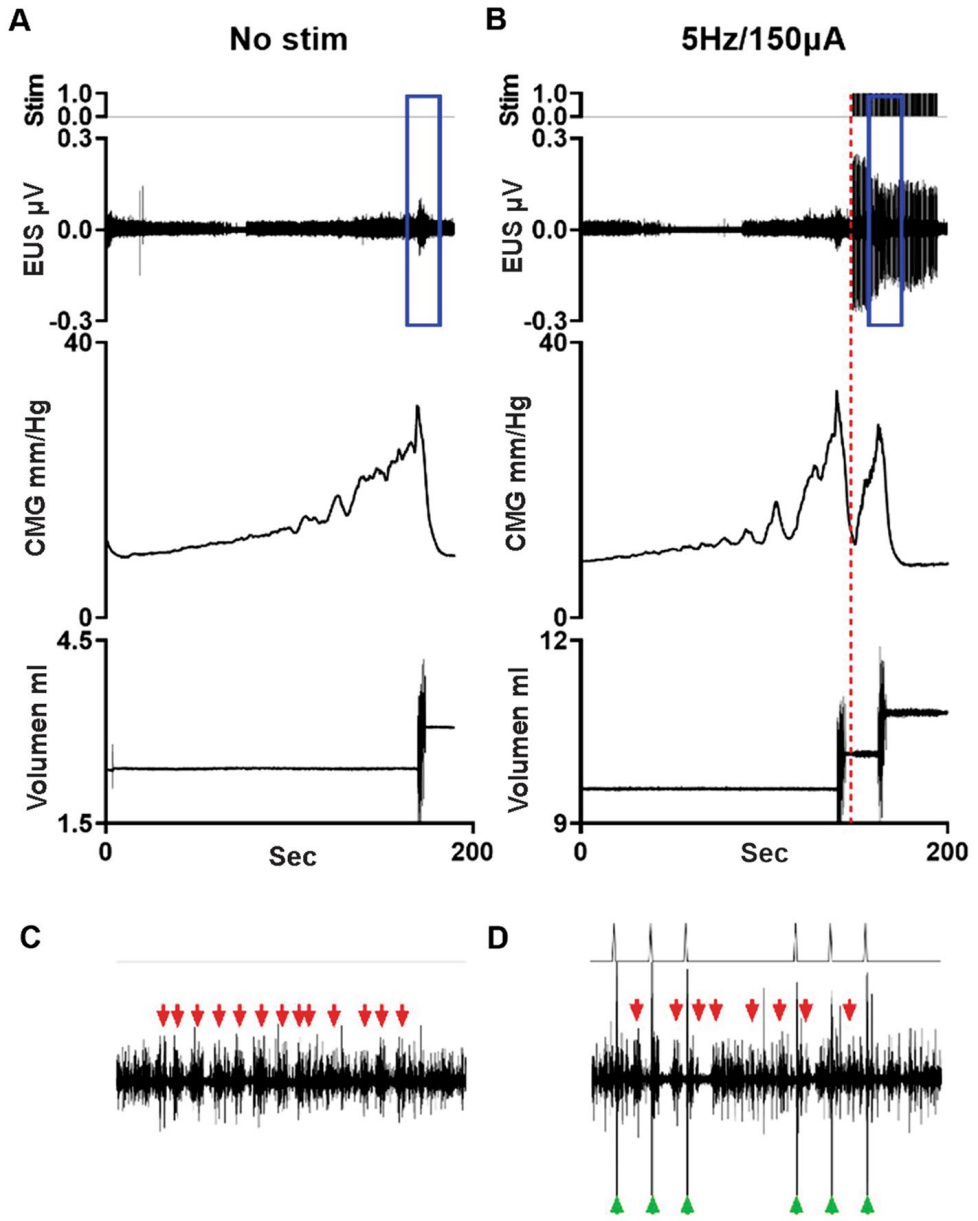
*Effect of scES at L3 in a partial burst subgroup rat.* The one transected animal having partial maintenance of burst activity at the chronic post-injury timepoint presented pre-scES with reduced compliance and some non-voiding contractions during the filling phase and large void volumes (**A**). scES at 150 μA for all frequencies triggered immediate and effective voids (**B**). The blue rectangles in A and B highlight the zoomed areas shown in (**C** and **D**). The phasic activity of EUS in SCI animals is characterized by irregular bursting pattern with an important reduction during the off periods. The scES at L3 level induced EUS activation (green arrows). However, the endogenous bursting activity (red arrows) remained active in this animal.

After chronic injury, differential outcomes were noted in response to L3 scES of each of the injury subgroups relative to the scES of shams. The main effect of L3 scES at 5 Hz/150μA in the burst subgroup was a reduction of the ICI (22.45 s), and a noticeable increase of the in–out ratio (r = 13.68) compared to baseline (r = 1.27) values (Fig. 5, Supplemental Table 2).

**Figure 6:**
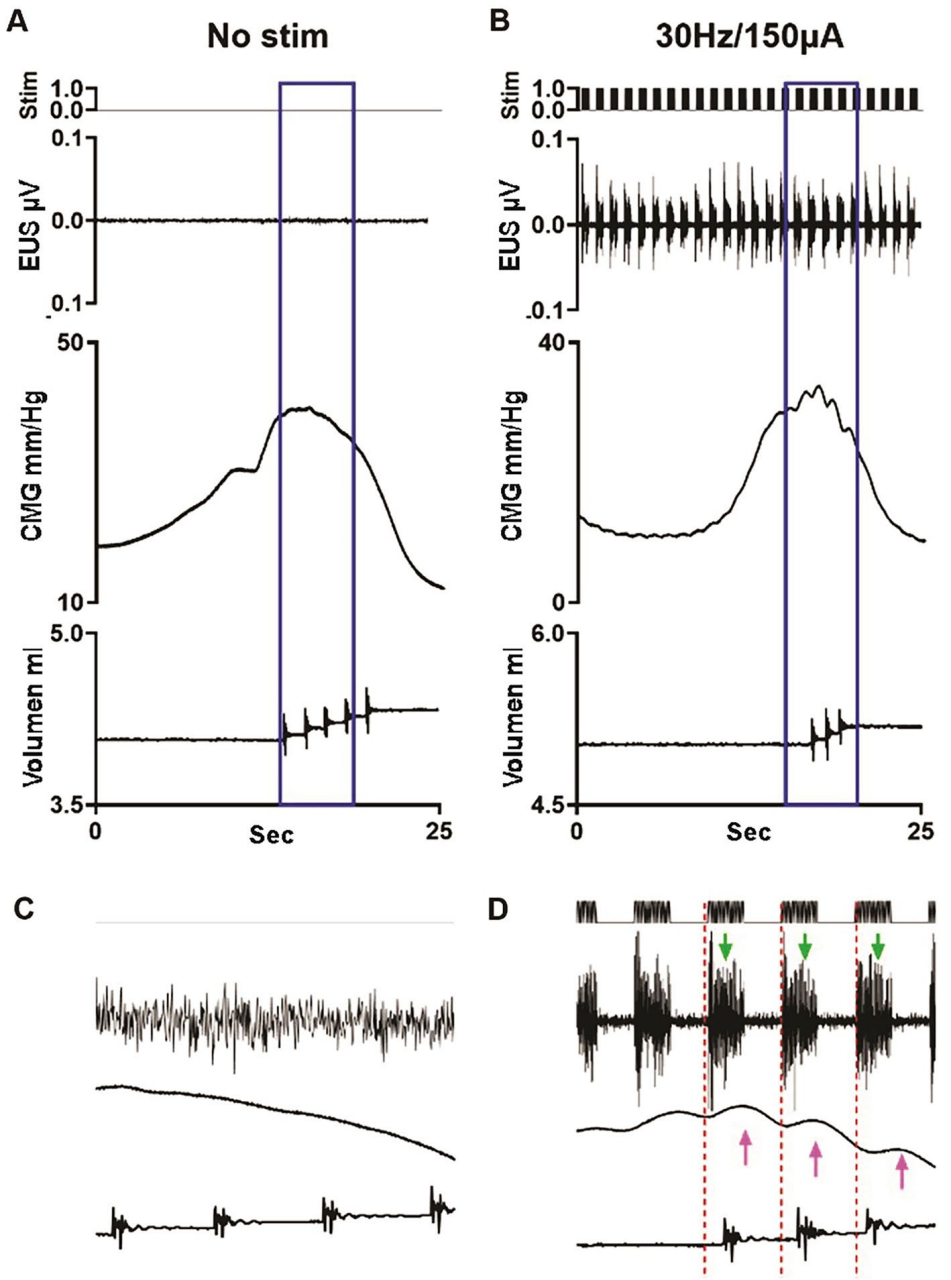
Effect of the scES at L3 in the detrusor sphincter dyssynergia (DSD)* subgroup.* SCI animals having DSD burst patterns presented with high bladder pressure prior to urine expulsion, tonic activity of the EUS during void, and reduced void volumes (**A**). Increasing scES intensities caused a gradual decrease in void volume, statistically significant at 5 Hz/300 μA (**B**)*.* The blue rectangles in (**A** and **B**) highlight the magnified peak contraction areas shown in (**C** and **D**). The phasic activity of EUS in SCI animals was characterized by tonic activity of the EUS and the absence of HFO’s. scES at L3 induced EUS activation (**D**, green arrows) and the presence of stimulus dependent HFO (magenta arrows) and thus expulsion of urine with every stimulus train. This induced CMG-EMG resembles the typical voiding pattern in sham animals with different burst frequency and duration.

**Figure 7:**
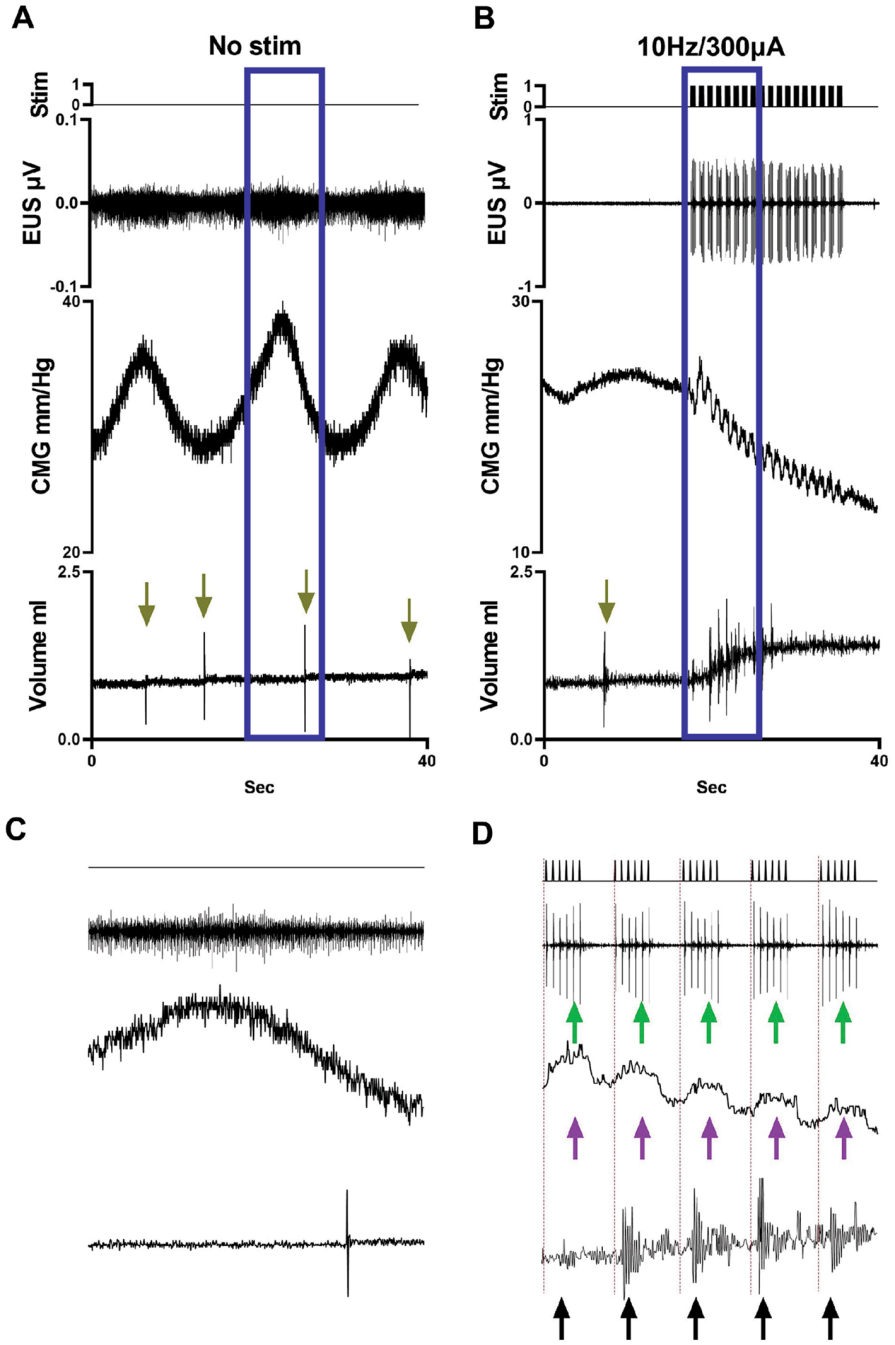
Effect of the scES at L3 in rats displaying overflow incontinence. The overflow subgroup had incontinence characterized by small contractile activity of the detrusor at very high basal pressure, constant EUS tonic activity and the expulsion of a single drop with every contraction (**A** and **C**). scES at 300 μA for all frequencies triggered an immediate and effective void (typical example shown in **B**). The blue rectangles in (**A** and **B**) highlights the magnified areas shown in (**C** and **D**). The scES at L3 level induced EUS activation (**D**, green arrows) and the presence of stimulus dependent HFO (magenta arrows) and thus expulsion of urine with every stimulus train. This induced CMG-EMG resembles the typical voiding pattern in sham animals with different burst frequency and duration.

**Figure 8:**
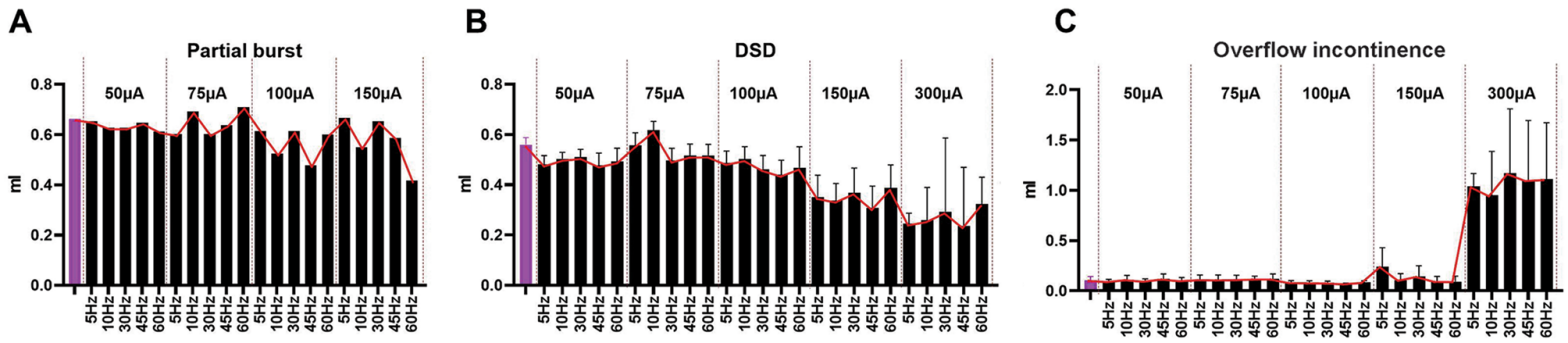
Summary of the combination of all frequencies and intensities for scES at L3 on the cystometric parameters within the different groups of injured female rats. The red lines in (**A** and **B**) indicate the trending reduction in void volumes with increasing intensities (partial burst and DSD subgroups). For the overflow incontinence subgroup (**C**), the trend (low N so not statistically significant) shows the increase in void volume at 300 μA for all frequencies.

In the DSD subgroup (typical example provided in Fig. 6), scES at high intensities (150 and 300μA) triggered an activation of the EUS where the duration of the activation matched the duration of the stimuli trains. These EUS contractions were reflected in the bladder pressure in the same manner as sham animals (Fig. 2A) and the expelled fluid had a temporal correspondence with stimulation and stimulated EUS contractions (Fig. 6D). In the overflow incontinence subgroup (Fig. 7), the scES at high intensity (300 μA) had similar effects to those observed in the DSD group, including activation of the EUS matched with the duration of the stimuli trains, the rhythmic increases on the bladder pressure, and the timing of the drops (Fig. 7D). In terms of voided volume (Fig. 8., Supplemental Table 2), scES trended toward a decrease at higher intensities in the DSD group and an increase in the overflow incontinence group (not statistically significant due to low subgroup N’s but trend is evident).

## Discussion

This study examined the effect of scES at the L3 spinal level on urinary bladder and EUS function in both uninjured and T9 spinally transected female rats. The absence of all supraspinal inputs to the lumbosacral circuits controlling LUT function after an anatomically complete transection maximizes the known neuroplasticity^24–27^ and subsequent potential changes in scES effects post-SCI relative to intact controls. Physiological parameters were collected by performing terminal urodynamic procedures in urethane anesthetized rats while applying different combinations of scES with varying intensity and frequency. The results of this mapping study show the effect of scES at the L3 level is highly dependent on the phenotypic urodynamic function prior to stimulation (I.e., presence of partial EUS bursting, DSD, or overflow incontinence), demonstrating the importance of the spinal circuits controlling voiding reflexes at this level^28,29^ in both uninjured and after complete chronic SCI^24–27^.

The activity of the EUS in rats has been extensively studied^30–32^ and shown to be crucial for efficient micturition due to the pumping action of the rhythmic contractions. Indeed, as seen with the typical example in Fig. 4D, scES at high frequencies and intensities results in phasic activation (with a similar frequency to scES trains) but disrupts the normal pattern of EUS bursting. Such a disruption negatively impacts the voided volume, and presumably the voiding efficiency (as estimated by the in–out ratio, Fig. 3A and D). These results highlight the importance of normal EUS bursting activity for efficient voiding in rats.

Intensity and frequency dependent increases in the ICI were found (Fig. 3B) indicating that certain combinations of scES parameters cause an inactivation of the normal bladder reflexes and therefore elongate the ICI. These data are consistent with previous studies which show that tonic activation of EUS, by scES at L3 level in uninjured animals^33^. Because recurrent inhibition of the sacral parasympathetic neurons has a positive relationship with increasing frequencies of stimulation^12^, scES is likely changing the ICI by modulating these parasympathetic pathways to promote higher bladder capacities.

Bursting activity in the EUS has been shown to be modulated by L3 scES^33^. Data from the current study shows a frequency dependent increase in the duration of burst activity (Fig. 3C) occurred during L3 scES. Previous data also shows that a higher in/out ratio is a consequence of longer EUS bursting periods^23^. The frequency dependent increase of the in–out ratio in this study is consistent with those observations (Fig. 2D).

Noteworthy is the observation of subgroups of animals showing different urodynamic outcomes post-transection which cannot be explained by the SCI itself (histological verification of an anatomically complete transection was conducted). The subgroups observed in this study are likely due to variable neuroplastic changes below the level of injury. Different functional outcomes in the chronic stage post-SCI could be due to a number of factors, including differences in post-injury bladder management, urinary tract infections, the presence of bladder stones, or other idiopathic post-injury factors, which can lead to structural changes in both the detrusor muscle and neural control^5,18,24,34,35^.

The three described patterns of urination following SCI have been described previously, but a clear understanding of the changes underlying these patterns is still an open question^15,16,24,30,31,36,37^. An added complication is that the previously described neural circuits controlling LUT function can vary and literature descriptions are sometimes contradictory^38^. Furthermore, the same level and grade of SCI can lead to different urinary complications in human patients^17,39,40^ indicating a more thorough understanding of the changes that occur after SCI is needed . In consideration of the above factors, we hypothesize that the neural plasticity occurring in the lumbo-sacral circuitry after SCI is not uniform and therefore can cause functional differences in individuals that have the same SCI injury characteristics. Thus, targeting these circuitries with a therapeutic intervention such as scES would require some degree of initial mapping to optimize functional outcomes across individuals. Furthermore, the surgical placement of the bladder catheter and/or EUS electrodes, could also impact urodynamic performance in SCI animals^23,41^.

As seen in the first subgroup of SCI rats where the EUS bursting is still present after spinal cord complete transection, scES at 5 Hz, and intensities above 100µA, triggers an immediate and effective void (Fig. 5B). As previously shown in spinally intact animals, electrical stimulation of Barrington’s nucleus triggers bladder contractions and urination^42,43^ through direct connections from the PMC to the sacral sympathetic neurons controlling the urinary bladder via the dorsolateral funiculus (DLF)^44^. Additionally, direct stimulation of the LSCC at L3 level causes bursting activity of the EUS^33^. Therefore, scES in this study is likely producing the observed effects in the first subgroup of animals (Bursting) by activation of the L3 LSCC structures^10,45,46^ where it is possible that the PMC projections modulate the bursting activity under normal conditions. Interestingly, the DLF is one of the principal targets for the pain-relieving effect of scES^47^ by activation of the endogenous opioid system which has also been shown to modulate urinary bladder activity^48,49^.

The results observed in the DSD group show the capacity of scES to rhythmically activate the EUS (Fig. 6B). The presence of a nuclear region, adjacent to the central canal within the gray matter, at the L3-L4 spinal segment that is responsible for controlling the phasic contractile activity of the EUS during voiding has been confirmed via physiologic, pharmacologic, molecular, and trans-neuronal tracing techniques^20,21,33,50^. Additionally, it has been shown that scES at L3-L4 segments can selectively modulate the activity of EUS, inactivating the tonic activity^33^, or triggering the phasic contraction^51^ by changing the characteristics of the electrical stimuli. Therefore, the scES applied in this study is likely changing LUT function by modulating the activity of LSCC in the DSD group.

Although the scES induced activation of the EUS resulted in a decrease in voided volume in this group of animals (Fig. 8B), the temporal matching between the EUS electrical activity, the HFO’s, and the releasing of a urine drop with each EUS activation (Fig. 6D) demonstrates the effectiveness of this scES-induced EUS activity. Intermittent activation of the EUS during a bladder contraction has been used successfully to treat DSD in people living with SCI via sacral anterior root stimulation^52^. In a similar way, the relaxation of EUS while the detrusor is still contracting explains the flow of urine during stimulation in this study. Importantly, intermittence of urine flow due to the rhythmic opening-closure of the EUS is the typical pattern of micturition in rats^32,38,53^. Therefore, these data support the hypothesis that physiologically relevant timing of frequency and duration of the scES pulses (mimicking the natural frequency, active-inactive ratio, and duration of the bursting activity) can improve the voided volume in SCI animals. Indeed, studies have shown reduced voiding efficiency from frequencies and durations of stimulation that alter the naturally occurring bursting of the EUS ^30,54^, which could be due to incomplete relaxation of EUS during stimulation, impairing urine flow.

The finding that some but not all female rats exhibited overflow incontinence upon reaching capacity (continuous fluid expulsion at an elevated detrusor pressure) is consistent with what was observed in our previous studies^15,16^ which found a higher prevalence in males versus females. This group of animals showed a mixed response to L3 scES, which consisted of bladder contraction (similarly to the burst subgroup) and the stimulus-dependent activation of the EUS (as with the DSD subgroup). Stimulation at L3 in this group triggered a massive expulsion of urine (Fig. 7B), which is consistent with scES at different spinal levels in our previous studies^15,16^ whereby the interconnected network within the lower cord^21,33,50^ is activated. Additionally, the stimulus-dependent bursting generated by the activation of the EUS motoneurons can explain the high voided volumes collected from these animals. The poor performance of the urinary bladder prior to scES makes this finding more relevant as it shows that the modulation of L3 centers, mimicking the physiological rhythmical intermittence of the EUS during the voiding phase of the cycle, can improve the voiding performance in SCI animals which could have enormous translational potential for the use of scES at the L3 level to treat humans with SCI-induced LUT dysfunction. Preliminary evidence from such studies by our group in humans supports this potential (unpublished observations).

Taken together, the results of this study demonstrate that scES at the L3 spinal level can modulate the activity of both the bladder and EUS in intact and SCI rats. This further highlights the L3 segment as a promising target for the treatment of DSD in SCI patients using scES. These data also show the importance of different frequencies and intensities of scES in the response to stimulation as demonstrated by changes in EUS bursting and the impact on voiding efficiency. Additionally, the identification of three distinct phenotypes of LUT dysfunction (partial burst, DSD and overflow incontinence) after complete transection, and specific responses to L3 scES of each subgroup, highlights the complexity of neuroplasticity during SCI recovery and reinforces the importance of a patient-centered evaluation of post-injury complications. Note that larger groups will be necessary in future studies as the post-study division of 7 SCI rats into different functional outcome sub-types reduced power (important trends revealed but not to the level of significance due to N’s of 1 and 2 in two of the subgroups). Importantly, the current findings illustrate the need for further studies of urinary complications in a variety of clinical scenarios to fully understand the complex autonomic changes that occur after SCI. Furthermore, the ability of L3 scES to activate the EUS, and generate artificial bursting activity, holds great potential for improving effective emptying for people living with SCI. The timing between bladder and sphincter as well as the heterogeneity of urinary functions post-SCI supports the need for a multi-modal neuromodulation approach.

## Supplementary Information

Supplementary Information.

## Data Availability

To request data for this study, contact Dr. Charles Hubscher at chhubs01@louisville.edu. As part of the NIH SPARC Materials Sharing policy, the curated datasets generated and/or analyzed for the current study will be available on the SPARC portal.
