# Supplementary material for: Mid-lumbar (L3) epidural stimulation effects on bladder and external urethral sphincter in non-injured and chronically transected urethane-anesthetized rats
Source: Sci Rep. 2023 Jul 28;13:12258. doi: 10.1038/s41598-023-39388-9 (PMC10382500; doi:10.1038/s41598-023-39388-9)
Supplement: Supplementary file 1 — Supplementary Information. [file 41598_2023_39388_MOESM1_ESM.pdf]

Supplement for:

Mid-lumbar (L3) epidural stimulation effects on bladder and external urethral sphincter in non-injured and chronically transected urethane-anesthetized rats.

Daniel Medina-Aguiñaga, Robert F. Hoey, Natasha Wilkins, Beatrice Ugiliweneza, Jason Fell, Susan J. Harkema, and Charles H. Hubscher.

Supplemental Table 1: Summary of CMG and EUS EMG Data at Baseline

	Surgical Shams N=8	Complete Spinal Transection		
		Partial Burst N=1	Detrusor Sphincter Dyssynergia N=4	Overflow Incontinence N=2
Inter-Contraction Interval (sec)	109.1 ± 40.9	159.38 ± 14.2	99.5 ± 28.83	29.9 ± 18.72
# Non-Void Contractions	0	3 ± 1	1.4 ± 1.2	0
Mean Urethral Opening Pressure (mmHg)	23.31 ± 5.69	31.9 ± 0.93	37.65 ± 4.34	34.34 ± 2.94
Mean Void Volume (ml)	0.53 ± 0.06	0.66 ± 0.04	0.54 ± 0.06	0.103 ± 0.05
In-Out Ratio	0.98 ± 0.22	1.01 ± 0.12	1.4 ± 0.2	0.86 ± 0.12
EUS # Bursts per Void	0	7.5 ± 0.86	0	0
EUS Burst Duration (sec)	3.2 ± 0.33	2.47 ± 0.77	0	0

Supplemental Table 2: Summary of CMG and EUS EMG Data at Optimal scES

	Surgical Shams 5Hz/300µA N=8	Complete Spinal Transection		
		Partial Burst 5Hz/150µA N=1	Detrusor Sphincter Dyssynergia 5Hz/300µA N=4	Overflow Incontinence 5Hz/300µA N=2
Inter-Contraction Interval (sec)	109.28 ± 47.51	22.45	74.5 ± 23.33	18 ± 14.14
Mean Urethral Opening Pressure (mmHg)	18.19 ± 4.95	28.6	33.05 ± 10.11	33 ± 1.5
Mean Void Volume (ml)	0.37 ± 0.31	1.28	0.26 ± 0.02	1.03 ± 0.17
In-Out Ratio	1.27 ± 1.75	13.68	0.86 ± 0.18	18.68 ± 12.35
EUS # Bursts per Void	25.2 ± 11.03	14	0	0
EUS Burst Duration (sec)	2.58 ± 4.58	4.53	0	0
